# Reply to Romano and De Dreu: Why violent extremism cannot be reduced to laboratory games

**DOI:** 10.1073/pnas.2613434123

**Published:** 2026-05-22

**Authors:** Jonas R. Kunst, Tomasz Besta, Milan Obaidi

**Affiliations:** ^a^ Department of Communication and Culture, BI Norwegian Business School, Oslo 0484, Norway; ^b^Institute of Psychology, University of Gdańsk, Gdańsk 80-309, Poland; ^c^ Department of Psychology, University of Copenhagen, 1353 Copenhagen, Denmark

We appreciate Romano and De Dreu’s commentary (1) concerning our recent article (2). While the intentions-behavior gap remains a pervasive challenge, their critiques misapprehend the unique psychological and ecological nature of violent extremism by conflating it with generic intergroup resource competition.

Our observed double dissociation is extremely difficult to attribute to payoff miscalculation or response bias. Social dominance orientation predicted offensive intentions nearly twice as strongly as defensive ones, while Machiavellianism showed the reverse pattern. If defensive prevalence were driven by payoff structure alone, traits reflecting dominance-seeking versus strategic manipulation should predict offense and defense similarly (since both would flow from the same cost–benefit calculus), yet they do not.

The commentators go on to suggest the higher prevalence of defensive intentions reflects strategic payoff considerations rather than moral legitimacy. However, these mechanisms are not mutually exclusive. Perpetrators often experience their violence as morally necessary (3). Our finding that Machiavellianism was most strongly associated with defensive intentions illustrates this: strategically manipulative individuals may exploit defensive framing as a reputational resource. Yet, under a pure payoff account, they should endorse whichever form of violence is situationally optimal. Their preference for the defensive frame seems therefore anomalous from a payoff perspective, and only coherent if moral legitimacy constitutes an independent motivational resource. Reducing this dynamic to pure payoff logic misses the powerful motivational narratives required to mobilize actual extremist violence.

Romano and De Dreu further argue that our findings are confounded by miscalibrated expectations about outgroup competitiveness. However, subjective threat perceptions are not a confound—they are the psychological reality that fuels the threat-security channel (4, 5). Perpetrators routinely frame their actions as defensive, reflecting a “hawkish bias” (6) of perceiving themselves as reacting.

The apparent contradiction between our finding that liberal identification predicts higher offensive extremist intentions and Romano and De Dreu’s new game-theoretic result that conservatives invest more in attack behaviors dissolves once the distinction between acquisitive and maintenance offense is applied—a taxonomy we explicitly introduced. Their “attack” behavior in laboratory games and our offensive intentions variable capture fundamentally different behavioral and motivational domains. The former indexes economic resource competition in neutrally framed monetary contests. The latter measures identity-driven, morally saturated intentions to enact group-dominance violence; these correlated with independent, behaviorally grounded macrolevel indicators of actual violence. By contrast, within our own taxonomy, the commentators’ attack behavior most plausibly captures maintenance offense (i.e., hierarchy enforcement and norm policing), which aligns with conservatism’s system-defending orientation. Our offensive scale measures acquisitive, status-quo-disrupting violence, a promotion-focused goal that can align with extreme liberals’ system-challenging collective action orientation.

Thus, Romano and De Dreu’s paradigm is not a valid proxy for group-based violent extremism. It involves individuals making small monetary decisions against opponents identified by nationality, using language neutralized to remove ideological or moral framing. It was designed to study economic competition, not the complex dynamics of violent extremism, where sacred values, identity fusion, and martyrdom willingness often structurally override standard utility calculations (7, 8).
